# Mitigating jaw osteonecrosis: bioactive glass and pericardial membrane combination in a rat model

**DOI:** 10.3389/fonc.2024.1348118

**Published:** 2024-05-10

**Authors:** Alexandre Antonio Pellicano, Bernar M. Benites, Amanda F. N. Paschoa, Laura C. Oliveira, Ana Carolina P. Campos, Daniel O. Martins, Caroline C. Real, Daniele de Paula Faria, Felipe P. Fonseca, Raquel R. C. Martinez, Rosana L. Pagano, Eduardo R. Fregnani

**Affiliations:** ^1^ Laboratory of Neuroscience, Hospital Sírio-Libanês, São Paulo, SP, Brazil; ^2^ Department of Radiology and Oncology, Faculdade de Medicina da Universidade de São Paulo, São Paulo, SP, Brazil; ^3^ Department of Oral Surgery and Pathology, School of Dentistry, Universidade Federal de Minas Gerais, Belo Horizonte, MG, Brazil; ^4^ LIM/23, Institute of Psychiatry, University of Sao Paulo School of Medicine, Sao Paulo, Brazil

**Keywords:** bisphosphonate-related osteonecrosis of the jaw, zoledronic acid, computed tomography, PET image, rat

## Abstract

**Objectives:**

Bisphosphonates (BFs) show clinical effectiveness in managing osteoporosis and bone metastases but pose risks of bisphosphonate-related jaw osteonecrosis (BRONJ). With no established gold standard for BRONJ treatment, our focus is on symptom severity reduction. We aimed to assess the preventive effects of bioactive glass and/or pericardial membrane in a preclinical BRONJ model, evaluating their potential to prevent osteonecrosis and bone loss post-tooth extractions in zoledronic acid (ZA)-treated animals.

**Methods:**

Rats, receiving ZA or saline biweekly for four weeks, underwent 1st and 2nd lower left molar extractions. Pericardial membrane alone or with F18 bioglass was applied post-extractions. Microarchitecture analysis and bone loss assessment utilized computerized microtomography (CT) and positron emission tomography (PET) with 18F-FDG and 18F-NaF tracers. Histological analysis evaluated bone injury.

**Results:**

Exclusive alveolar bone loss occurred post-extraction in the continuous ZA group, inducing osteonecrosis, osteolysis, osteomyelitis, and abscess formation. Concurrent pericardial membrane with F18 bioglass application prevented these outcomes. Baseline PET/CT scans showed no discernible uptake differences, but post-extraction 18F-FDG tracer imaging revealed heightened glucose metabolism at the extraction site in the ZA-treated group with membrane, contrasting the control group.

**Conclusion:**

These findings suggest pericardial membrane with F18 bioglass effectively prevents BRONJ in the preclinical model.

**Translational potential of this article:** The imperative for novel therapeutic strategies aimed at facilitating alveolar bone repair remains paramount in the prevention of BRONJ subsequent to tooth extraction. Our investigation underscores that the combination of pericardial membrane and bioglass exhibits promising translational potential in the prevention of BRONJ.

## Introduction

Bisphosphonates (BPs) represent a class of medications employed in the management of a spectrum of medical conditions, including malignant bone lesions such as bone metastasis from malignant neoplasms, malignant hypercalcemia, and multiple myeloma, as well as metabolic bone diseases such as osteoporosis and Paget’s disease (1, 2). Generally, these drugs are well-tolerated and rarely cause clinically significant side effects. Oral BPs may induce gastrointestinal symptoms, while injectable BPs may lead to elevated serum creatinine, transient low fever, arthralgias, and increased bone pain (3). Nonetheless, it should be noted that prolonged usage of these medications has been linked to a significant complication referred to as bisphosphonate-related osteonecrosis of the jaw (BRONJ), however, it is imperative to acknowledge that patients should be classified as being at risk from the initiation of medication use (4, 5). Given the focus of this study on bisphosphonate-induced osteonecrosis, we shall employ the term “BRONJ.” Nevertheless, it is worth noting that the broader term “MRONJ” (Medication-Related Osteonecrosis of the Jaw) is presently accepted when discussing osteonecrosis induced by medications, particularly antiresorptive and antiangiogenic agents (6). BRONJ typically occurs when the alveolar bone in the jaw fails to heal following a tooth extraction, resulting in exposed bone and the development of an infectious focus (7). The prevalence of BRONJ in oncology patients receiving high doses of oncology doses of BPs ranges from 1% to 15%, while in patients with osteoporosis receiving a low dose of BPs, it is reported to be between 0.001% and 0.01% (8).

Since its initial report nearly two decades ago, there has been an increasing number of publications focused on BRONJ, and some progress has been made in understanding its pathophysiology mechanisms (9, 10). However, significant knowledge gaps still remain. One approach to addressing these gaps is the collaborative effort of research groups aiming to improve preclinical models that replicate the development of BRONJ (11). Some studies have applied preclinical models to simulate the treatment of patients with ZA (12) and have demonstrated the effects of BPs on bone tissue following tooth extraction (13, 14). Nevertheless, the limited understanding of BRONJ’s pathogenesis greatly hampers its prevention and treatment, and a standardized protocol has yet to be established.

Several reports have been published on the effects of adjuvant therapies primarily used for prevention, including photobiomodulation (15), hyperbaric oxygen therapy (16), and platelet-rich plasma therapy (17). However, an effective protocol has not been established thus far. In this investigation, we proposed the utilization of biomaterials, notably bioactive glass, in conjunction with an acellular membrane made from bovine pericardium, as an innovative approach to furnish structural reinforcement to compromised alveolar bone, thus expediting the process of bone repair subsequent to tooth extraction.

Biocompatible materials are widely used to induce various biological responses upon contact with tissues or physiological fluids (18). F18 bioactive glass, in particular, exhibits a broader range of functionalities compared to other bioactive glasses (19) and has the capacity to promote the formation of new bone and soft tissue (20). Pericardium membrane is another biomaterial that has been successfully employed in numerous surgical procedures, including dural grafting (21), hernia treatment (22), and coating orbital implants after enucleation (23). The use of a high-resistance bovine pericardium acellular membrane may offer benefits in preventing BRONJ; however, its effectiveness in preventing BP-induced osteonecrosis after tooth extraction has not been tested.

The main objective of this study was to validate a preclinical model of BRONJ in rats and investigate the preventive effects of F18 bioglass and/or pericardial membrane when applied immediately after extraction. Microarchitecture analysis and assessment of bone loss were conducted using computerized tomography (CT) and positron emission tomography (PET) for small animals with 18F-fluorodeoxy glucose (18F-FDG) or 18F-sodium fluoride (18F-NaF) tracers, and bone injury was evaluated through histological analysis. To the best of our knowledge, this is the first experimental study to investigate the effects of F18 bioglass and/or pericardial membrane treatment in preventing the development of BRONJ.

## Materials and methods

### Experimental design

**Figure 1:**
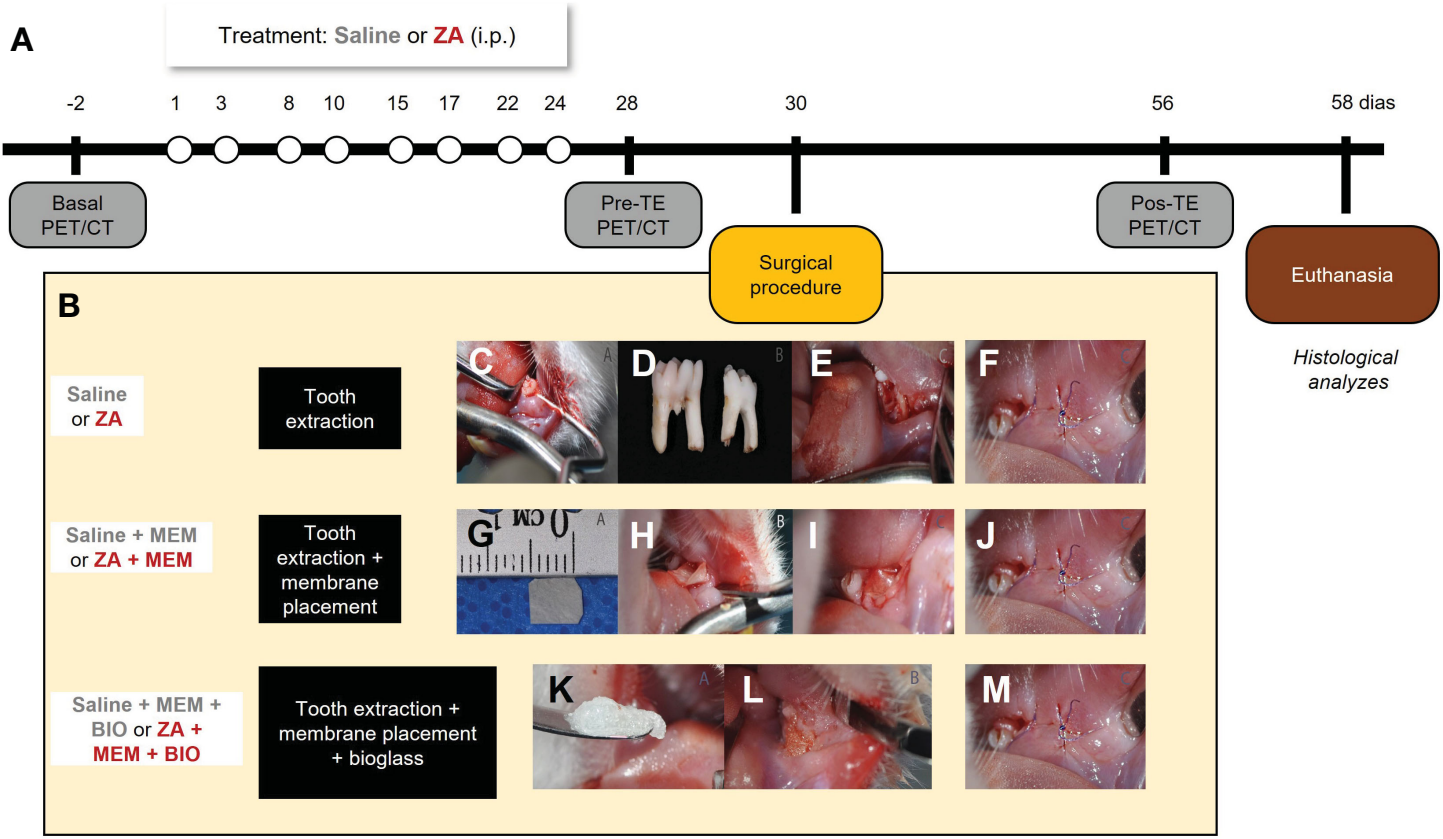
Experimental design **(A)**. Representative images of the surgical procedure and interventions **(B)**. Stages of the surgical extraction procedure. Positioning the instrument on the distobuccal cervical margin for dislocation of the dental element **(C)**. First and second molars extracted from Wistar rats **(D)**. Mandibular alveolus in the region of the first and second molars after tooth extractions **(E)**. Stages of adjuvant bioactive membrane therapy. Cutout of TechGraft bovine pericardium acellular biological membrane (5 mm x 5 mm) **(G)**. Beginning of membrane positioning in the dental socket in the vestibular epithelium of the left mandible **(H)**. Complete positioning of the pre-suture membrane **(I)**. Stages of adjuvant therapy with bioglass. Bioglass F18 **(K)**. Insertion of Bioglass F18 into the mandibular socket after extraction **(L)**. Suture in the region of the first and second lower left molars after extraction and presence of absence of biomaterial **(F, J, M)**.

Rats underwent small-animal PET imaging using 18F-FDG and 18F-NaF tracers. CT images were captured immediately afterward. A baseline *in vivo* PET/CT was acquired on anesthetized animals two days before ZA treatment to establish a comparison reference (Baseline image). On day 1 (D1), ZA treatment began, given twice a week for 4 weeks. Controls received saline under the same conditions. On day 28, another PET/CT acquisition was conducted to assess medication-related osteonecrosis before tooth extractions (PreTE). On day 30 (D30), tooth extractions were conducted as a trigger for osteonecrosis induction, followed by the application of biomaterials (either membrane placement or membrane placement combined with bioglass) and subsequent suturing. Imaging was carried out on day 56 (PostTE), with euthanization taking place on day 58 (D58). Mandibles were dissected for histological analysis. Experimental groups were (1): Saline (S) group: saline injections, extraction, suturing (n=3) (2); ZA group: ZA treatment, extraction, suturing (n=3) (3); ZA + membrane (ZA/MEM) group: ZA treatment, extraction, pericardial membrane, suturing (n=5); (4) Saline + membrane (S/MEM) group: saline injections, extraction, pericardial membrane, suturing (n=5); (5) ZA + bioglass + membrane (ZA/BIO/MEM) group: ZA treatment, extraction, pericardial membrane, bioglass, suturing (n=5); and (6) Saline + membrane + bioglass (S/MEM/BIO) group: saline injections, extraction, pericardial membrane, bioglass, suturing (n=5) (**Figure 1**).

### Animals

This was a controlled-blind study where evaluators remained unaware of the animals’ experimental groups. Twenty-six male Wistar rats (Rattus norvegicus albinus), aged 9 weeks and weighing 220 to 250 grams, were used. Rats were housed in acrylic boxes with three per box for over a week before the experiments. They were kept in controlled conditions (12/12 h light/dark cycle, 22 ± 2°C) with access to water and rat chow pellets. Animal procedures followed ARRIVE guidelines (http://www.nc3rs.org.uk/arrive-guidelines) and were approved by the Ethics Committee on Animal Use at Hospital Sírio-Libanês (Sao Paulo, Brazil), protocol CEUA 2018-01. The number of animals in each group was different to personalize each experiment. This approach was to ensuring that the experimental conditions were optimized for each individual case and to reduce the number of animals used.

### Establishment of a rat BRONJ model

The preclinical model of BRONJ was established following a previously described method (11). Rats were intraperitoneally treated with 200 μg/kg of ZA (Zometa, Novartis Biosciences S.A., Basel, Switzerland) twice a week for 4 weeks. The control animals received intraperitoneal injections of 0,9% saline solution (1.5 mL) twice a week for 4 weeks. Subsequently, the animals were anesthetized with isoflurane (4% induction, 2.5% maintenance in 100% oxygen) and local infiltration anesthesia (2% lidocaine, 200 µl/site) was administered to mitigate intra- and postoperative pain. Representative photomicrographs of the surgical procedure can be found in **Figure 1B**. The extraction of the lower left 1st and 2nd molar was performed using a dental explorer probe (SS White, RJ, Brazil), as previously described (24). The instrument’s tip was positioned at the disto-vestibular gingival margin of each tooth, and the operator applied rotational movements to detach the gingival and periodontal fibers (**Figure 1C**). Then, the instrument was positioned in the furcation region to extract the teeth, and any interdental septa were removed. For control animals (Saline and AZ without biomaterial application), the alveoli were sutured with 8-0 suture threads (Vicryl-Poliglactina 910-Ethicon, SP, Brazil) immediately after tooth extraction (**Figures 1D–F**).

### Application of biomaterials

After tooth extractions, animals that received the membrane (ZA + membrane and saline + membrane), the alveoli were covered with a bioactive bovine pericardium acellular membrane (Techgraft, Baumer S.A., SP, Brazil, **Figure 1G**) before suturing (**Figures 1H–J**). In animals that received the membrane combined with the bioglass (ZA + membrane + bioglass and saline + membrane + bioglass groups), the alveoli were initially filled with F18 bioglass (VETRA, SP, Brazil, **Figure 1K**) diluted in 0.9% saline solution. Subsequently, the alveoli were covered with the pericardial membrane (**Figure 1L**) and sutured (**Figure 1M**).

### Postoperative care

To ensure the well-being of the animals after dental extractions, the rats were observed for signs of bleeding around the mouth and excessive swelling outside the operated area. After the extractions, the rats were given sodium dipyrone (100 mg/kg) every 12 hours under the skin for the first 72 hours to manage immediate pain. Additionally, their weekly water and food intake, along with their weight, were measured. If the rats lost 20% of their post-extraction weight, the experiment would be stopped prematurely, and the rats would be euthanized.

### Positron emission tomography/computed tomography (PET/CT) analysis

Rats were scanned using a small-animal PET/CT equipment (Triumph Trimodality, Gamma Medica-Ideas, CA, USA) with 18F-FDG and 18F-NaF, followed by the acquisition of CT images immediately after.

### PET

Animals were anesthetized with 5% isoflurane in oxygen and maintained on 2-3% isoflurane in O2 during the injection of the radiopharmaceutical and throughout the image acquisition. While the animals were under anesthesia, a blood sample was obtained from the tail to measure blood glucose levels by Accu-check Active glucometer (Roche Diagnostics GmbH). The radiopharmaceutical, 18F-FDG or 18F-NaF (37- 48 MBq in up to 1 mL), was administered intravenously into the penile vein of the anaesthetized animals. After the injection, the animals were allowed to wake up from anesthesia to facilitate the even distribution of the radiopharmaceutical throughout their bodies. After 45 minutes for 18F-FDG and 60 minutes for 18F-NaF injections, the animals were anaesthetized again and positioned with their heads in the center of the small animal PET scanner’s field of view and images acquired for 30 minutes. Images of 18F-FDG and 18F-NaF were acquired on different (subsequent) days to account for radioactive decay, ensuring that one radiopharmaceutical does not interfere with the image of the other. The animals were kept warm, and their heartbeats monitored throughout the time the images are acquired. PET images were reconstructed using the 3D ordered subsets expectation maximization (OSEM) algorithm. After the PET images were acquired, the animals were repositioned for computed tomography (CT) imaging using the same equipment, with 1-minute acquisition using 45 kVp and 400 µA and magnification of 1.3 times. CT images were reconstructed using the filtered back projection (FBP) algorithm. The purpose of the CT images was to provide anatomical reference for fusion with the PET images. The analysis of the PET images was performed using PMOD software version 4.1 (PMOD Technologies, Switzerland). The uptake of radiotracers was presented as a standardized uptake value (SUV), calculated as the radioactivity concentration (kBq/cc) divided by the ratio of the injected dose (kBq) to animal’s body weight (g).

### CT

**Figure 2:**
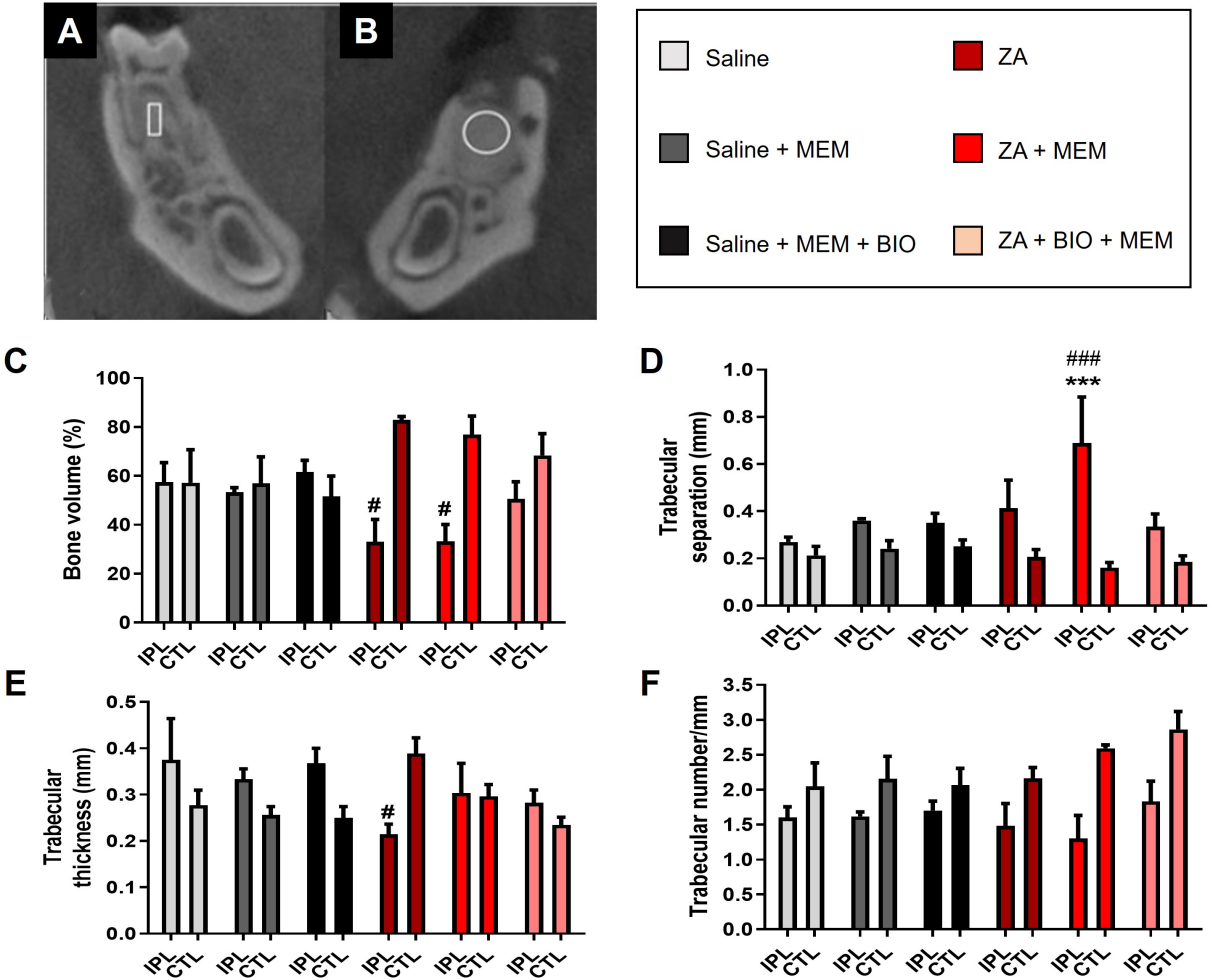
Evaluation of computed tomography for small rodents (CT). Three-dimensional analysis (3D) CT cross-sections indicate the region of interest in the two hemimandibles. Assessment of alveolar bone structure **(A)**. Evaluation of bone repair of the dental socket **(B)**. Quantification, obtained by CT, of the of bone volume **(C)**, trabecular separation **(D)**, trabecular thickness **(E)** and number of trabeculae **(F)** and trabecular separation of saline (n=3), saline + membrane (n= 5), saline + bioglass + membrane (n=5), ZA (n=3), ZA+ membrane (n=5) and ZA + bioglass + membrane (n=5). Statistical analysis Two-way ANOVA, ***p<0.001 when compared to saline-treated animals. ^#^p < 0.05; ^###^p < 0.0001 when compared to the CTL side. IPL, side ipsilateral to extraction; CTL, contralateral side to extraction.

Immediately after the PET acquisition and anatomic CT acquisition, a magnified CT was acquired to evaluate alveolar bone loss in the mandibles and bone matrix during repair. A CT scan was performed with 256 projections, 45 kVp, 400 µA, and a magnification factor of 3. CT reconstruction was carried out using the filtered back projection (FBP) algorithm. Multiplanar reconstructions (axial, sagittal, and coronal) were created using AMIRA software (Zuse Institute, Berlin, Germany). The “volume of interest” (VOI), representing a three-dimensional (3D) measurement, was evaluated based on the ROIs and slice thickness. Following CT imaging, the animals were returned to their home cage. For the 3D evaluation, the DataViewer software (SkyScan, Kontich, Belgium) was used. A parallelepiped-shaped geometric figure was created to define the volume of interest (10 μm wide, 30 μm high, and consisting of 11 slices with a thickness of 40 μm). A parallelepiped was positioned in the central area between the roots of the first molar to assess the volume of interest in the right hemimandible. The right hemimandible was used as a control to evaluate the alveolar bone structure, considering that no tooth extraction was performed (**Figure 2A**). Moreover, using the same DataViewer software, a cylindrical-shaped geometric figure was created to assess another volume of interest (40 μm in diameter and composed of 11 slices with a thickness of 40 μm). The cylindrical geometric shape was positioned in the center of the alveolus of the distal root of the first molar in the left hemimandible (**Figures 2A, B**). The characteristics of trabecular bone microarchitecture evaluated in the right and left hemimandibles were: (a) bone volume fraction (bone volume/total volume); (b) trabecular separation (mm); (c) trabecular thickness (mm) and (d) trabecular number per mm, following a previously proposed methodology (25).

### Histological processing

On day 58, the animals were euthanized using an anesthetic overdose of isoflurane, and their mandibles were dissected and placed in a 10% formalin solution. After 24 hours, the mandibles were decalcified in 4% EDTA for 30 days. Subsequently, they underwent conventional histological processing and were embedded in paraffin blocks. Histological sections with a thickness of 5 μm were stained with hematoxylin and eosin for histological analysis. Microscopic images for histological analysis were captured using a 200x objective of a photomicroscope (Leica Biosystems, Nussloch, Germany). The histological sections were blindly analyzed by two researchers using a conventional optical microscope, aiming to identify specific parameters such as gingival ulceration, osteonecrosis, osteomyelitis, osteolysis, presence of bacterial colonies, and bone neoformation. The presence or absence of each of the parameters was recorded, and the data were presented as the percentage of occurrence. In the event of differences of opinion during the analysis, the evaluators reached a consensus to determine the presence or absence of the observed parameter.

### Data analysis

The sample size was calculated based on preliminary data regarding the differences observed in bone volume (%) and trabecular thickness (mm) between saline and ZA treated animals (**Supplementary Figure 1**) as previously described (26). Results were expressed as mean ± SEM. Data were analyzed with GraphPad Prism (CA, USA). For PET and CT evaluation, two-way analysis of variance (ANOVA) followed by Tukey’s multiple comparison *post hoc* test was performed. For the presence of histological findings, Chi-Square was performed. In all cases, p < 0.05 were considered statistically significant. The data obtained from the CT technique were subjected to quantitative analysis to assess the extent of bone loss, as well as qualitative analysis to evaluate the pattern of bone loss. Linear evaluations were performed using Amide^®^ software, while volumetric evaluations utilized the DataViewer^®^ software. The results of the CT analysis were presented as mean and standard deviation and compared using the Student’s t-test for independent measures with IBM SPSS Statistics 22.0 (IBM Corp, Armonk, NY, USA). Statistical analyses for the baseline data obtained from PET imaging were conducted using one-way Analysis of Variance (ANOVA). Following the surgical procedures, data were analyzed using two-way ANOVA, followed by a Tukey’s *post hoc* correction for multiple comparisons. Histologic analysis was employed to examine changes in the cellular structures of the mandible. The results are presented as the percentage of occurrence for each parameter. For these analyses, the two-way ANOVA test was used, with factor 1 being laterality and factor 2 being group, or one-way ANOVA followed by Tukey’s *post hoc* test. Statistical significance was set at 5% (α = 0.05).

## Results

### Characterization of preclinical BRONJ model

First, we aimed to understand the effect of ZA treatment upon tooth extraction in the absence of any biomaterial (**Supplementary Figure 1A**). When comparing saline and ZA animals not submitted to any biomaterial during the surgical procedure, we found that ZA-treated animals had a decreased bone volume (F_(1,2)_ = 22.32, p = 0.04, **Supplementary Figure 1B**) and trabecular thickness (F_(1,2)_ = 9.32, p = 0.01; **Supplementary Figure 1D**) when compared to saline treated animals and contralateral mandible. Interestingly, ZA animals showed a non-significant increase of 80% in trabecular separation (F_(1,2)_ = 12.41, p = 0.07; **Supplementary Figure 1C**) and no difference regarding trabecular number F_(1,2)_ = 0.06, p = 0.82, **Supplementary Figure 1D**).

**Figure 3:**
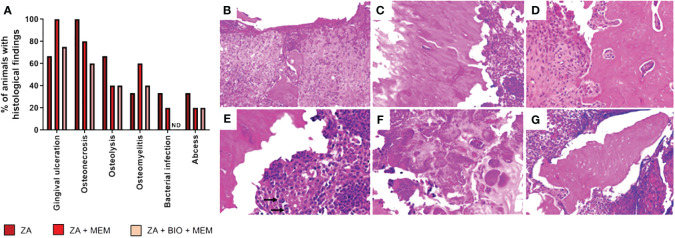
Histological analyses. Effect of biomaterials on the histological findings observed in the region of tooth extractions in the preclinical model of osteonecrosis. Evaluation of the percentage **(A)** of attendance of gingival ulceration, osteonecrosis, osteolysis, osteomyelitis, bacterial colony, abscess, and bone formation in animals treated with ZA followed by extraction (ZA, n=3), animals treated with ZA followed by membrane immediately after extraction (ZA+MEM, n =5) and animals treated with ZA followed by bioglass combined with membrane right after tooth extraction (ZA+BIO+MEM, n=5). Characterization of the histological findings. **(B)** Gingival ulceration: Loss of surface epithelium, with areas of hemorrhage and chronic inflammatory infiltrate (Hematoxylin and Eosin - HE; 100X). **(C)** Osteonecrosis: Necrotic bone, characterized by the presence of empty lacunae (HE; 200X). **(D)** Osteolysis: Presence of multinucleated giant cells (osteoclasts) adjacent to necrotic bone (HE; 200X). **(E)** Osteomyelitis: Chronic inflammatory infiltrates associated with necrotic bone; arrows show osteoclasts cells (HE; 200X). **(F)** Bacterial colony: Presence of bacterial colonies (HE; 400X). **(G)** Abscess: Non-vital bone fragment surrounded by inflammatory cells forming areas of an abscess (HE; 200X). ND, not detected.

Histological analysis revealed ZA-treated animals displayed conditions like gingival ulceration (66%), osteonecrosis (100%), osteolysis (66%), osteomyelitis (33%), bacterial colonies (33%), and abscess formation (33%) after tooth extractions, which were absent in saline-treated animals (**Supplementary Figure 1F**). Nevertheless, both groups exhibited bone formation. ZA-treated rats exhibited features like gingival ulceration, osteonecrosis, osteolysis, osteomyelitis, bacterial colonies, and abscess formation. These findings confirm the validity of the preclinical BRONJ model. **Figure 3** illustrates the histological aspects of gingival ulceration (**Figure 3B**), osteonecrosis (**Figure 3C**), osteolysis (**Figure 3D**), osteomyelitis (**Figure 3E**), bacterial colony (**Figure 3F**), and abscess (**Figure 3G**).

### Effect of the use of biomaterials in the preclinical BRONJ model

Regarding baseline and PreTE values, no statistically significant difference was observed among the experimental groups in terms of bone volume (F_(5, 18)_ = 0.88, p = 0.6; **Supplementary Figure 2**). To assess the effectiveness of bioglass and/or pericardial membrane in preventing observed events in the preclinical BRONJ model, animals treated with saline or ZA received these materials immediately after molar extractions.

As previously shown, ZA treatment decreased bone volume and trabecular thickness when compared to saline-treated animals and the contralateral mandible. Interestingly, only the combination of bioglass and periodontal membrane was able to prevent the loss of bone volume (F_(5, 19)_ = 16.38, p = 0.0007; **Figure 2C**) when compared to controls. The use of biomaterials, either the membrane alone or bioglass combined with the membrane, immediately after tooth extractions prevented the reduction in trabecular thickness observed in ZA-treated animals after dental extractions (F_(5.19)_ = 5.24, p = 0.003; **Figure 2E**). No significant differences were noted between the groups regarding the number of trabeculae (F_(5.19)_ = 1.071, p = 0.40; **Figure 2F**). Interestingly, ZA-treated animals that received the membrane after dental extractions displayed an exacerbated increase in trabecular separation compared to the contralateral side and other groups (F_(5.19)_ = 3.006, p = 0.036; **Figure 2D**).

In terms of histological analysis, ZA-treated animals receiving the pericardial membrane immediately after tooth extractions exhibited gingival ulceration (100%), osteonecrosis (80%), osteolysis (40%), osteomyelitis (60%), bacterial colonies (20%), and abscesses (20%). Animals treated with ZA receiving bioglass combined with the pericardial membrane immediately after tooth extractions displayed gingival ulceration (75%), osteonecrosis (60%), osteolysis (40%), osteomyelitis (40%), and abscesses (20%), with none of the animals in this group showing bacterial colonies (**Figure 3A**).

In 18F-FDG PET images, no significant difference in SUV ratios was found among groups in baseline, preTE, and postTE analyses. The SUV baseline ratios were consistent across groups. Similarly, no significant differences were found in SUV ratios in preTE and postTE images among groups.

In 18F-NaF PET images, no statistically significant difference in SUV ratios was observed among the groups (p = 0.638). However, the SUV ratios showed differences between groups in preTE and postTE images. In the postTE images, statistically significant differences were observed, particularly comparing the ZA/BIO group with the control group. (**Tables 1**, **2**) provide more detailed data on SUV ratios in both 18F-FDG and 18F-NaF PET images.

**Table 1:** Results of 18F-FDG PET at baseline, pre and post-tooth extraction.

Treatments		AZ + Salin	AZ + Mem	Salin + Memb	AZ+Memb+Bio	Sal+Memb+Bio
Ipsilateral	Baseline	1,06 ± 0,04	0,96 ± 0,15	0,88 ± 0,07	1,08 ± 0,14	1,12 ± 0,13
	Pre	1,02 ± 0,15	0,95 ± 0,17	0,87 ± 0,07	1,20 ± 0,27	0,92 ± 0,10
	Pos	0,86 ± 0,06	0,84 ± 0,05	1,03 ± 0,06	1,13 ± 0,09	1,15 ± 0,25
Contralateral	Baseline	1,02 ± 0,10	0,97 ± 0,17	0,91 ± 0,09	1,06 ± 0,06	1,09 ± 0,10
	Pre	1,05 ± 0,13	0,93 ± 0,24	0,82 ± 0,05	1,22 ± 0,30	0,91 ± 0,10
	Pos	0,89 ± 0,09	0,84 ± 0,17	0,99 ± 0,16	1,06 ± 0,28	0,95 ± 0,20

Tracer uptake in the ipsilateral (experimental) and contralateral (control jaw, expressed as Standard Uptake Value (SUV). Data are expressed as Mean ± SD.

**Table 2:** Results of 18F-NAF PET at baseline, pre and post-tooth extraction.

Treatments		AZ + Salin	AZ + Mem	Salin + Memb	AZ+Memb+Bio*	Sal+Memb+Bio
Ipsilateral	Baseline	4,85 ± 2,29	5,97 ± 0,49	6,22 ± 0,16	6,59 ± 0,66	6,63 ± 0,60
	Pre	4,24 ± 1,56	5,63 ± 0,53	6,52 ± 0,40	5,83 ± 1,23	6,94 ± 0,47
	Pos	5,70 ± 1,63	5,81 ± 1,32	7,28 ± 0,79	5,18 ± 0,65	7,37 ± 0,22
Contralateral	Baseline	4,95 ± 2,51	5,94 ± 0,56	6,17 ± 0,43	6,49 ± 0,81	6,75 ± 0,44
	Pre	4,19 ± 1,77	5,64 ± 0,59	6,54 ± 0,28	5,87 ± 1,33	7,05 ± 0,60
	Pos	5,61 ± 1,25	5,28 ± 1,47	7,30 ± 0,68	4,69 ± 0,55	7,29 ± 0,70

Tracer uptake in the ipsilateral (experimental) and contralateral (control) jaw, expressed as Standard Uptake Value (SUV). Data are expressed as Mean ± SD. *Only the group AZ+Memb+Bio was statistically significantly different from ipsi and contralateral injured sides (p = 0,0336) but wasn’t different from the other groups (p = 0,0639).

## Discussion

In our study, continuous ZA treatment led to significant alveolar bone loss and a range of complications, including osteonecrosis, osteolysis, osteomyelitis, and abscess formation. However, the application of pericardial membrane combined with F18 bioglass post-tooth extraction effectively prevented these adverse events, with distinctive changes observed in glucose metabolism in the ZA-treated group.

The emergence of BRONJ as a complication in patients on anti-resorptive and/or anti-angiogenic treatments is noteworthy. However, there is divergence among studies regarding the incidence and prevalence of this condition (27). ZA, a widely used medication in medical practice, is particularly important due to its potent anti-resorptive and antitumor properties, making it a commonly used therapy for oncologic patients (28). BRONJ can lead to prolonged recovery, impairing oral health functionally (29). Dentoalveolar surgeries and periodontal diseases are the most frequently associated local risk factors for BRONJ (29). Tooth extraction models with ZA treatment have been established for BRONJ (11, 30). In this study, we adopted the model described by Soundia and collaborators (11) to replicate osteonecrosis-like lesions of the jaws. They reported a high prevalence of up to 75% in the region of post-extraction dental alveoli under intraperitoneal administration of ZA, with tooth extraction being the trigger for the osteonecrosis process (11). This finding is consistent with the existing literature, which highlights osteonecrosis of the jaws as a severe adverse effect of BP therapy and recommends avoiding surgical procedures like tooth extraction to minimize the risk of BRONJ (1, 29). Our study replicated BRONJ characteristics with ZA treatment twice a week for 4 weeks, followed by tooth extraction, resulting in reduced alveolar filling, bone volume, and trabecular thickness. ZA treatment followed by extraction induced osteonecrosis, osteolysis, osteomyelitis, bacterial colonies, and abscesses, solidifying the preclinical BP-induced osteonecrosis model.

PET/CT functional imaging showed the accumulation of 18F-FDG and 18F-NaF in the tooth-affected BRONJ. Quantitative regional analysis of NaF-PET and FDG-PET scans confirmed an increased standardized uptake value (SUV) in the ipsilateral BRONJ areas. Interestingly, despite the treatments, we observed an enhancement in bone quality.

CT analysis is considered the gold standard for evaluating the microarchitecture of mineralized tissues. Parameters such as bone volume fraction (bone volume/total volume), thickness, number of trabeculae, and trabecular separation are commonly used to investigate pathological conditions in bone (31). In a study by Muller et al. (32), CT was validated as an evaluation method for measuring and analyzing cancellous bone in 3D. These authors compared the morphometric results of conventional histomorphometry with CT results and found an excellent correlation between the measured indices of trabecular bone architecture in the 3D representation compared to conventional 2D histology (33).

A variable degree of bone loss was observed in the quantitative analysis of linear measurements. Our study demonstrated that tooth extractions in animals treated with ZA were a significant factor in the development of osteonecrosis. The evaluations of baseline CT scans and pre-extractions scans in animals treated with saline or ZA showed that bone changes were restricted to animals undergoing extractions, which reinforces the role of extractions and other clinical surgical procedures as risk factors for the development of BRONJ, as suggested in previous studies (33). However, these evaluations also suggest changes in 3D bone architecture and decreased bone remodeling capacity due to the use of ZA, leading to a higher risk of BRONJ (33). It is important to note that there are limitations to the interpretation of our data, mainly due to the insufficient number of animals used in our study, which prevents reaching definitive conclusions.

There is strong interest in finding preventive treatments that can aid in bone repair, aiming to alleviate the sequelae of osteonecrosis and promote a faster recovery of the stomatognathic system. This is essential to ensure the proper functioning of essential activities in the body, as well as to restore the individual’s quality of life and social interaction (34). In this context, our qualitative analysis of the histological sections revealed that animals pretreated with saline without a biomaterial, as well as those treated with only the membrane or with bioglass combined with the membrane, showed normal bone repair. This was characterized by the formation of mature bone tissue within the alveolus, observed in most of the animals and covered by the oral mucosa epithelium. Our findings are consistent with the literature, which demonstrated that bone filling can be clinically observed at the end of the healing process after tooth extraction (35, 36). It is worth noting that animals pretreated with saline without biomaterial and those that received only the membrane after extraction showed gingival ulceration without significant bone repair. On the other hand, most animals pretreated with ZA that received the membrane and the combined use of membrane and bioglass immediately after extraction showed a delay in alveolar bone repair, characterized by the presence of areas of osteonecrosis and gingival ulceration. These findings are consistent with other studies that have evaluated the effects of ZA on bone repair in dental alveoli in rats (37, 38). Bone neoformation was a common finding in all cases, which is supported by previous research (39). However, the presence of bone neoformation alone does not directly correlate with bone quality and function, and therefore, it lacks functional significance.

The histological evaluations performed quantitative analyses for various variables including osteonecrosis, gingival ulceration, osteolysis, bacterial colony, osteomyelitis, abscess, and bone formation. It was observed that the presence of the membrane increased the prevalence of gingival ulceration and osteomyelitis. However, the combination of bioglass with the membrane was more effective in inhibiting the prevalence of osteonecrosis. Similarly, the presence of the membrane alone inhibited the prevalence of osteolysis and abscess, which was consistent with the effects observed with the combination of biomaterials. None of the animals pretreated with ZA and received bioglass combined with membrane showed bacterial colony formation. The absence of bacterial colonies in the animals receiving these biomaterials confirms the high broad-spectrum bactericidal activity of F18 bioglass against various bacterial strains (40). F18 bioglass also showed anti-biofilm activity when applied as a coating on implants (41). The results of the present study align with the findings reported by other research groups regarding the bioactivity and bactericidal effect of F18 bioglass (40).

It was observed that animals treated with ZA and animals treated with ZA that received membrane shortly after extraction showed a decrease in bone volume at the lesion site. However, animals treated with ZA that received bioglass combined with the membrane did not show a difference in bone volume when comparing the sides with and without extraction. This suggests that F18 bioglass was able to prevent bone loss and promote a good bone repair process after extraction. Furthermore, the use of biomaterials, whether it was the membrane alone or bioglass combined with the membrane, prevented the reduction of trabecular thickness, indicating a preventive function in the promotion of trabecular bone neoformation post-extraction in ZA-treated animals.

These findings expand our knowledge by demonstrating that it is possible to prevent the BRONJ process using the combination of bioglass and membrane, thus improving patients’ quality of life. This study is the first to show the benefits of F18 bioglass in combination with a bioactive acellular membrane of bovine pericardium as a therapeutic approach in the prevention of BRONJ. As limitations of the study, it should be noted that CT images were solely used for morphometric assessments, and the inflammatory profile of the gingival epithelium was not evaluated due to the loss of this lining epithelium in some animals during surgery.

This study sheds light on the promising potential of the bioglass and membrane combination as a preventive strategy for BRONJ, promising to enhance the quality of life for affected patients. It stands as the pioneering work to underscore the advantageous effects of F18 bioglass in conjunction with a pericardial membrane in the prevention of BRONJ. Moreover, the establishment of a preclinical model for BP-induced osteonecrosis and the demonstration of favorable preventive outcomes against BRONJ-like lesions underscore the significance of combining bioglass and membrane as a management approach for BRONJ. This research holds promise in shaping future clinical interventions to safeguard patients from this debilitating condition.

## Data availability statement

The raw data supporting the conclusions of this article will be made available by the authors, without undue reservation.

## Ethics statement

The animal study was approved by Hospital Sírio-Libanês protocol CEUA 2018-01. The study was conducted in accordance with the local legislation and institutional requirements.

## Author contributions

AP: Conceptualization, Investigation, Methodology, Validation, Writing – original draft. BB: Investigation, Methodology, Writing – original draft. AFP: Investigation, Methodology, Writing – original draft. LO: Investigation, Methodology, Writing – original draft. AC: Conceptualization, Data curation, Formal Analysis, Methodology, Writing – original draft, Writing – review & editing. DM: Data curation, Formal Analysis, Writing – original draft, Writing – review & editing. CR: Investigation, Methodology, Writing – review & editing. DF: Investigation, Methodology, Writing – review & editing. FF: Investigation, Methodology, Writing – review & editing. RM: Conceptualization, Data curation, Methodology, Validation, Writing – review & editing. RP: Conceptualization, Data curation, Formal Analysis, Funding acquisition, Methodology, Project administration, Resources, Supervision, Validation, Visualization, Writing – review & editing. EF: Conceptualization, Data curation, Formal Analysis, Funding acquisition, Project administration, Resources, Supervision, Validation, Visualization, Writing – review & editing.

## Supplementary material

Supplementary Figure 1 Experimental design **(A)**. Evaluation of computed tomography (CT). Three-dimensional analysis (3D) CT cross-sections indicate the region of interest in the two hemimandibles. Quantification, obtained by CT, of the of bone volume **(B)**, trabecular separation **(C)**, trabecular thickness **(D)** and number of trabeculae **(E)** and of saline (n=3) and ZA (n=3) animals. Statistical analysis Two-way ANOVA, *p<0.05 when compared to saline-treated animals. IPL: side ipsilateral to extraction. CTL: contralateral side to extraction. Histological analyses. Effect of biomaterials on the histological findings observed in the region of tooth extractions in the preclinical model of osteonecrosis. Evaluation of the percentage **(F)** of attendance of gingival ulceration, osteonecrosis, osteolysis, osteomyelitis, bacterial colony, abscess, and bone formation in animals treated with saline or ZA followed by extraction. ND, not detected.

Supplementary Figure 2 Evaluation of computed microtomography (CT). Three-dimensional analysis (3D) CT cross-sections indicate the region of interest in the two hemimandibles. Quantification, obtained by CT, of the of bone volume of saline (n=3), saline + membrane (n= 5), saline + bioglass + membrane (n=5), ZA (n=3), ZA+ membrane (n=5) and ZA + bioglass + membrane (n=5) comparing basal and pre-tooth extraction values. Statistical analysis Two-way ANOVA followed by Tukey’s post-hoc test. TE, tooth extraction.
