# Supplementary figures and images for: Mitigating jaw osteonecrosis: bioactive glass and pericardial membrane combination in a rat model
Source: Front Oncol. 2024 May 10;14:1348118. doi: 10.3389/fonc.2024.1348118 (PMC11116668; doi:10.3389/fonc.2024.1348118)

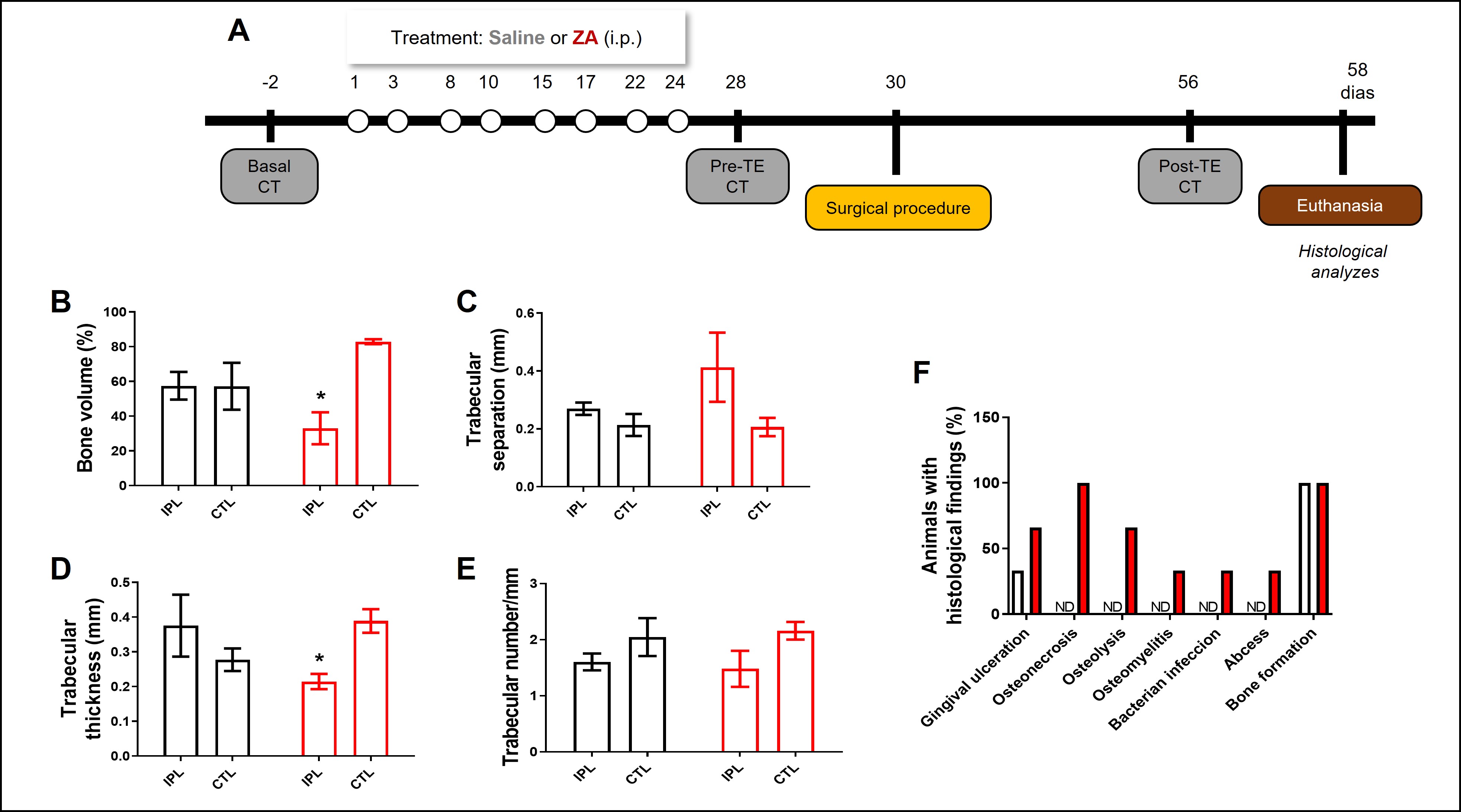

Supplement: Supplementary Figure 1 — Experimental design (A). Evaluation of computed tomography (CT). Three-dimensional analysis (3D) CT cross-sections indicate the region of interest in the two hemimandibles. Quantification, obtained by CT, of the of bone volume (B), trabecular separation (C), trabecular thickness (D) and number of trabeculae (E) and of saline (n=3) and ZA (n=3) animals. Statistical analysis Two-way ANOVA, *p<0.05 when compared to saline-treated animals. IPL: side ipsilateral to extraction. CTL: contralateral side to extraction. Histological analyses. Effect of biomaterials on the histological findings observed in the region of tooth extractions in the preclinical model of osteonecrosis. Evaluation of the percentage (F) of attendance of gingival ulceration, osteonecrosis, osteolysis, osteomyelitis, bacterial colony, abscess, and bone formation in animals treated with saline or ZA followed by extraction. ND, not detected. [file Image_1.jpeg]

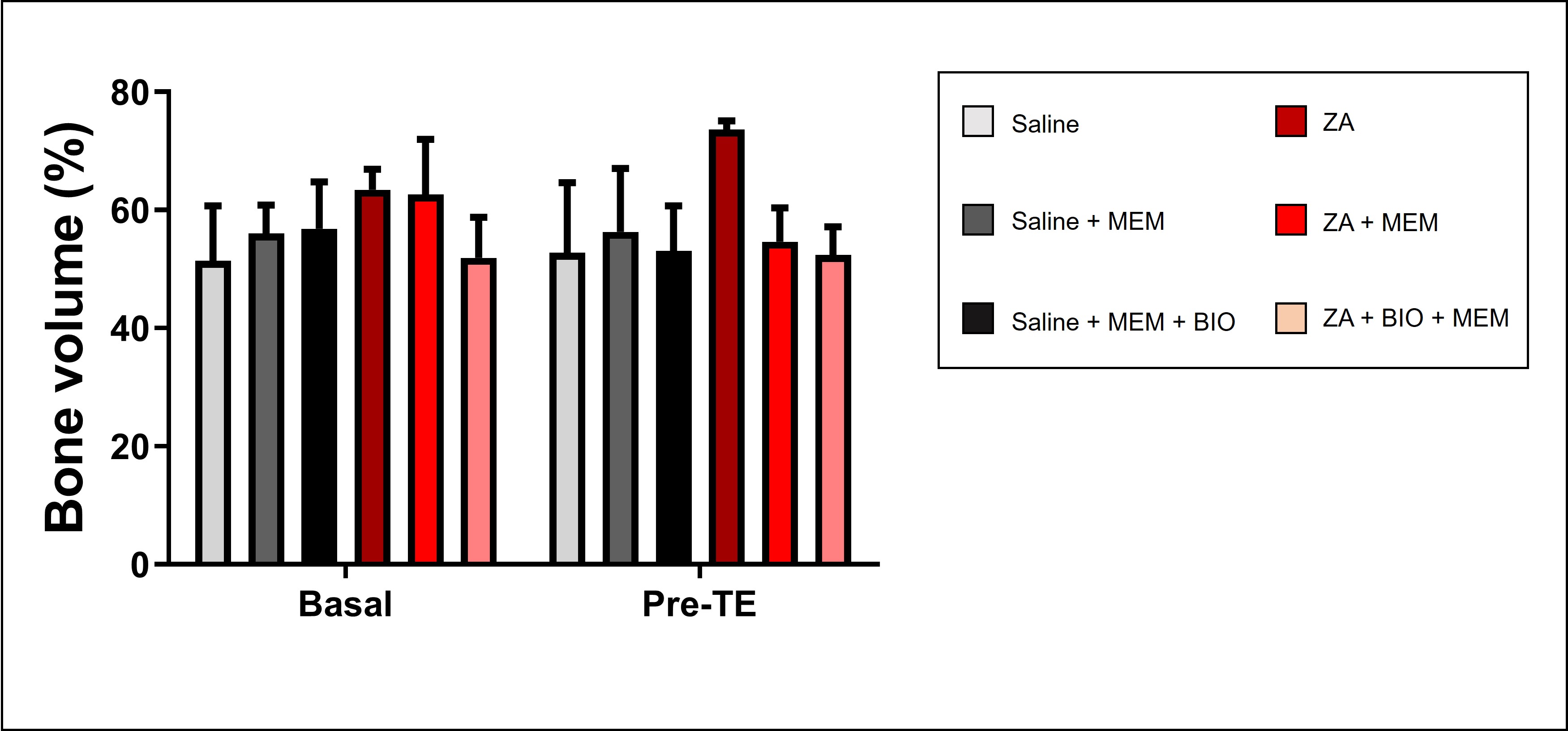

Supplement: Supplementary Figure 2 — Evaluation of computed microtomography (CT). Three-dimensional analysis (3D) CT cross-sections indicate the region of interest in the two hemimandibles. Quantification, obtained by CT, of the of bone volume of saline (n=3), saline + membrane (n= 5), saline + bioglass + membrane (n=5), ZA (n=3), ZA+ membrane (n=5) and ZA + bioglass + membrane (n=5) comparing basal and pre-tooth extraction values. Statistical analysis Two-way ANOVA followed by Tukey’s post-hoc test. TE, tooth extraction. [file Image_2.jpeg]
